# Assessing impulse control behaviors in early Parkinson’s disease: a longitudinal study

**DOI:** 10.3389/fneur.2023.1275170

**Published:** 2023-10-25

**Authors:** Xiaobo Zhu, Jing Gan, Na Wu, Ying Wan, Lu Song, Zhenguo Liu, Yu Zhang

**Affiliations:** Department of Neurology, Xinhua Hospital Affiliated to Shanghai Jiao Tong University School of Medicine, Shanghai, China

**Keywords:** Parkinson’s disease, impulse control behaviours, longitudinal assessment, dopamine transporter imaging, biomarkers

## Abstract

**Objective:**

Impulse control behaviors (ICBs) frequently coexist with Parkinson’s disease (PD). However, the predictors of ICBs in PD remain unclear, and there is limited data on the biological correlates of ICBs in PD. In this study, we examined clinical, imaging, and biological variables to identify factors associated with longitudinal changes in ICBs in early-stage PD.

**Methods:**

The data for this study were obtained from the Parkinson’s Progression Markers Initiative, an international prospective cohort study that evaluates markers of disease progression in PD. We examined clinical, imaging, and biological variables to determine their associations with ICBs over a period of up to 5 years. Cox regression models were employed to investigate the predictors of ICBs in early-stage, untreated PD.

**Results:**

The study enrolled 401 individuals with PD and 185 healthy controls (HC). At baseline, 83 PD subjects (20.7%) and 36 HC (19.5%) exhibited ICBs. Over the course of 5 years, the prevalence of ICBs increased in PD (from 20.7% to 27.3%, *p* < 0.001), while it decreased in HC (from 19.5% to 15.2%, *p* < 0.001). Longitudinally, the presence of ICBs in PD was associated with depression, anxiety, autonomic dysfunction, and excessive daytime sleepiness (EDS). However, there was no significant association observed with cognitive dysfunction or motor severity. Treatment with dopamine agonists was linked to ICBs at years 3 and 4. Conversely, there was no association found between ICBs and presynaptic dopaminergic dysfunction. Additionally, biofluid markers in baseline and the first year did not show a significant association with ICBs. A predictive index for ICBs was generated, incorporating three baseline characteristics: anxiety, rapid eye movement sleep behavior disorder (RBD), and p-tau levels in cerebrospinal fluid (CSF).

**Conclusion:**

During the early stages of PD, there is a notable increase in ICBs over time. These ICBs are associated with depression, anxiety, autonomic dysfunction, EDS, and the use of dopaminergic medications, particularly dopamine agonists. Anxiety, RBD, and p-tau levels in CSF are identified as predictors for the incident development of ICBs in early PD. Further longitudinal analyses will provide a more comprehensive understanding of the associations between ICBs and imaging findings, as well as biomarkers. These analyses will help to better characterize the relationships and implications of these factors in the context of ICBs in early PD.

## 1. Introduction

While Parkinson’s disease (PD) is primarily defined by its motor manifestations, it is important to note that non-motor symptoms (NMS) are also prevalent and can significantly impact an individual’s quality of life. These non-motor symptoms include impulse control behaviors (ICBs), which can be particularly detrimental (1). ICBs are characterized as repetitive, excessive, and compulsive abnormal behaviors that are driven by a strong desire and prove challenging to self-control (2, 3). Impulse Control Disorders (ICDs) represent the more severe manifestations of ICBs and encompass four specific types: pathological gambling (PG), hypersexuality (HS), compulsive buying (CB), and binge eating (BE). Furthermore, ICBs encompass additional related behaviors such as excessive hobbyism, punding, walkabout, and dopamine dysregulation syndrome (DDS) (3–5). Furthermore, as research progresses, the clinical spectrum of ICBs is expected to expand further. Recent studies have reported that newly relevant behaviors, such as over-donation and over-indulgence in mobile devices, may be included within the scope of ICBs (6–8).

Several studies have reported a wide variation in the prevalence of PD-ICBs, ranging from 3.5% to 59.0%. However, it is important to note that the majority of these studies were conducted on patients with intermediate to advanced stages of the disease who were already undergoing drug treatment (9–15). The mechanisms underlying PD-ICBs are currently unknown, and multiple factors have been implicated. These factors include being male, unmarried, younger age at the onset of PD, longer disease duration, certain medications (such as dopaminergic agonists, levodopa, amantadine, and rasagiline), personal or family history of smoking, drug or alcohol abuse, cultural factors (specifically residing in the United States), depression, anxiety, cognitive impairment, rapid eye movement sleep behavior disorder (RBD), restless legs syndrome (RLS), and genetic factors (3, 5, 15–19).

While numerous cross-sectional studies have investigated ICBs in PD, there is a limited number of studies that have examined their longitudinal incidence and prevalence. Furthermore, only a few studies have followed ICBs longitudinally for more than 5 years. The ICARUS study aimed to address this gap by examining longitudinal changes in the occurrence of ICBs over a 2-year period. The findings from this study indicated that the presence of ICBs remained relatively stable between the initial visit and the 2-year follow-up visit (20). However, several studies have reported a higher incidence of ICBs in early PD populations, and this incidence tends to increase over time (11, 14, 21). In a longitudinal study, the prevalence of ICDs was found to increase from 19.7% at baseline to 32.8% after a period of 5 years (21). Additionally, a study reported that ICBs were observed in 21 (19.8%) patients with PD and this prevalence increased to 29.2% at year 5 (11). In another study, ICBs were found in 38 (30.6%) patients with PD, and this prevalence significantly increased to 46.8% after a 4-year period (14). The variations in these findings can be attributed to the differences in the studied populations and the assessment methods employed. However, it is worth noting that previous studies have seldom reported the impact of biomarkers, such as cerebrospinal fluid (CSF) markers, on the prevalence of ICBs in newly diagnosed and untreated individuals with PD. Considering the potential association between PD-ICBs and biomarkers, investigating this aspect could hold substantial significance (2, 5, 20).

Given the existing knowledge gaps, our objective was to conduct a systematic investigation to provide a more comprehensive understanding of the prevalence, clinical spectrum, longitudinal evolution over a 5-year period, and biological correlates of ICBs in PD. To accomplish this, we utilized the Parkinson’s Progression Markers Initiative (PPMI) cohort. Additionally, we sought to assess the baseline biological factors that could potentially predict the development of ICBs in PD. By addressing these research objectives, we aimed to contribute valuable insights into the understanding and characterization of ICBs in PD, ultimately enhancing our knowledge of this important aspect of the disease.

## 2. Methods

### 2.1. Study design and participants

All the data utilized in this study were obtained from the PPMI database, which has been previously published and is accessible on the PPMI website^1^ (22). The PPMI study received approval from the institutional review board at each study center, and all participants provided signed written informed consent. The data utilized in this paper were derived from the baseline and 5-year follow-up dataset, which was downloaded on August 29, 2021.

During the screening process, individuals with PD were required to meet the following criteria: (1) exhibit at least two of the following: resting tremor, bradykinesia, and rigidity, or have an asymmetric resting tremor or asymmetric bradykinesia; (2) have received an idiopathic PD diagnosis within the past 2 years and remain untreated; (3) be aged 30 years or older; (4) undergo a screening dopamine transporter SPECT scan that demonstrates a dopamine transporter deficit. Regarding the healthy controls (HC), the following criteria were applied: (1) match PD participants in terms of age, gender, and education; (2) exhibit no significant neurological dysfunction; (3) demonstrate no cognitive impairment, as assessed by a Montreal Cognitive Assessment (MoCA) score of greater than 26; (4) have no family history of PD.

### 2.2. Study outcomes

To evaluate PD-ICBs, we employed the validated short version of the Questionnaire for Impulsive-Compulsive Disorders in Parkinson’s Disease (QUIP-S), a widely recognized and extensively validated tool recommended for screening ICBs in individuals with PD. The QUIP-S has been proven effective over time and is considered a reliable assessment instrument for this purpose (23). The scale comprises eight items that pertain to ICDs such as PG, HS, CB, and BE, as well as other behaviors including excessive hobbyism, punding, walkabout, and DDS. Consistent with previous studies, the presence of symptoms related to ICBs was defined as a score of ≥1 on any of the eight items. If a patient exhibits a combination of multiple symptoms simultaneously, it is considered as having multiple ICBs (24).

Furthermore, demographic and clinical data were collected for all subjects. Motor symptoms and disease severity were assessed using the Movement Disorders Society Unified Parkinson’s Disease Rating Scale (MDS-UPDRS) (25) and Hoehn and Yahr stage (H&Y) (26) respectively. These measures provide a comprehensive evaluation of motor symptoms and the overall severity of the disease in Parkinson’s patients. The MDS-UPDRS was used to calculate both the tremor score and the Postural Instability Gait Disorder (PIGD) score simultaneously. Additionally, the ratio of these scores was utilized to classify patients as having tremor-dominant (TD) or non-tremor-dominant (non-TD) subtypes (27). Additional assessments of NMS included the use of the Modified Schwab and England Activities of Daily Living Scale (S&E) (28), MoCA (29), the 15-item Geriatric Depression Scale (GDS) (30), the State–Trait Anxiety Inventory (STAI) state and trait subscores (31), the Epworth Sleepiness Scale (ESS) (32), the REM Sleep Behaviour Disorder Screening Questionnaire (RBDSQ) (33), the Scales for Outcomes in Parkinson’s Disease-Autonomic (SCOPA-AUT) (34). In this study, participants were considered to have a positive screening for RBD if they scored ≥5 on the RBDSQ (35). Dopaminergic therapy usage was quantified using the levodopa equivalent daily dose (LEDD), calculated according to a previously described method. The LEDD provides a standardized measure for comparing the dosage of different dopaminergic medications by converting them to an equivalent dose of levodopa (36).

We also conducted 123-I Ioflupane dopamine transporter (DaTscan) imaging to assess the dopamine transporter in all subjects. The analysis of the DaTscan images was performed according to the relevant manuals available at http://ppmi-info.org/ (22). Biological sample tests included measurements of serum urate, neurofilament light chain (NfL), and CSF analysis of A-beta 1–42, total tau (T-tau), tau phosphorylated at threonine 181 (P-tau181), and alpha-synuclein. Detailed information regarding sample collection, processing, and analysis can be found in the previously published reports related to this study (37).

### 2.3. Statistical analysis

All statistical analyses were conducted using SPSS 26. The t-test or chi-square test was used to compare baseline demographics and clinical characteristics between PD subjects and controls, as well as to compare demographics, clinical characteristics, DaTscan measures, and medication use at each time point between patients with PD with and without ICBs. Mann–Whitney U tests were employed to compare biologics between patients with PD with and without ICBs. T-test for normally distributed data, Mann–Whitney U tests for non-normally distributed data, and chi-square for categorical variables.

Logistic mixed models were employed to examine changes in ICB-related characteristics over time in patients with PD and HC separately. These models were also used to assess differences in ICBs between the two groups over time. In the latter models, an interaction term between visit and groups was initially tested to evaluate potential differential effects over time. If the interaction test did not reach statistical significance at the 0.10 level, the interaction term was removed from the model, and the overall group differences were reported.

In addition, Cox regression models were utilized to explore the univariate and multivariable relationships between baseline demographic, clinical, biological, imaging, and sedative use predictors and the prevalence of ICBs in PD, as well as their predictive value for changes over a 5-year period. To account for covariance, a specific scheme was adopted. For the DaTscan variables, if either the contralateral or ipsilateral side of the putamen or caudate measure exhibited statistical significance in univariate analysis, the contralateral side of the measure was prioritized for inclusion in the multivariate model. Likewise, for CSF biomarkers and the CSF ratios, only the biomarker was included in the multivariate model if both the specific biomarker and its associated ratio were found to be significant. CSF ratios were considered in the multivariate model only if there were instances where neither of the two biomarkers reached significance, but the CSF ratio showed significant associations. Lastly, plot Nomogram based on Cox result for better representation.

## 3. Results

### 3.1. Impulse control behaviours over time in PD and HC

**Figure 1:**
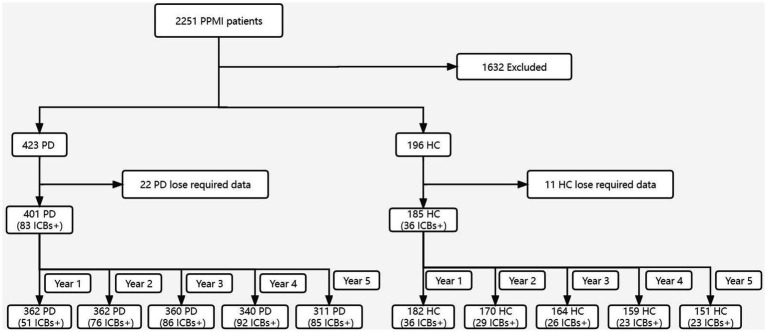
Flowchart of the participant selection. ICBs, impulse control behaviours; PD, Parkinson disease; HC, healthy controls; PPMI, Parkinson’s Progression Markers Initiative.

Figure 1 presents an overview of the sample selection process. Initially, data was obtained from 423 PD subjects and 196 HC. However, after thorough assessment, it was determined that only 401 PD subjects and 185 HC possessed all the required data. Consequently, for PD participants, data was accessible for 401 individuals at baseline, 362 individuals at year 1, 362 individuals at year 2, 360 individuals at year 3, 340 individuals at year 4, and 311 individuals at year 5. As for HC, data was available for 185 participants at baseline, 182 participants at year 1, 170 participants at year 2, 164 participants at year 3, 159 participants at year 4, and 151 participants at year 5.

Table 1 provides an overview of the baseline demographics of the cohort. It indicates that there were no significant differences observed between PD and HC groups in terms of demographics, including gender, age, and education.

**Table 1:** Baseline demographics and PD characteristic.

Variable	PD subjects			HCs subjects	*p* Value	*p* Value
	*n* = 423	PD ICBs+ *n* = 87	PD ICBs− *n* = 335	*n* = 196	(PDvs HC)	(ICBs + *vs* −)
QUIP-S					0.616	N/A
Positive (≥1)	87 (20.62%)	N/A	N/A	37 (18.88%)		
Negative (<1)	335 (79.38%)	N/A	N/A	159 (81.12%)		
Missing	1	N/A	N/A	0		
Gender					0.771	0.779
Male	277 (65.48%)	56 (64.37%)	221 (65.97%)	126 (64.29%)		
Female	146 (34.52%)	31 (35.63%)	114 (34.03%)	70 (35.71%)		
Missing	0	0	0	0		
Age (years)					0.319	0.284
Mean (SD)	61.69 (9.72)	60.66 (10.33)	61.91 (9.52)	60.81 (11.23)		
(Min, Max)	(34.00, 85.00)	(36.00, 83.00)	(34.00, 85.00)	(31.00, 84.00)		
Missing	0	0	0	0		
Age at PD onset (years)					N/A	0.225
Mean (SD)	59.70 (9.98)	58.51 (10.94)	59.96 (9.68)	N/A		
(Min, Max)	(25.00, 83.00)	(25.00, 81.00)	(30.00, 83.00)	N/A		
Missing	0	0	0	N/A		
Disease duration (month)					N/A	0.602
Mean (SD)	6.57 (6.49)	6.85 (7.01)	6.44 (6.31)	N/A		
(Min, Max)	(0.00, 36.00)	(1.00, 36.00)	(0.00, 35.00)	N/A		
Missing	0	0	0	N/A		
Education (years)					0.057	0.480
Mean (SD)	15.56 (2.97)	15.36 (2.79)	15.61 (3.02)	16.04 (2.89)		
(Min, Max)	(5.00, 26.00)	(5.00, 20.00)	(5.00, 26.00)	(8.00, 24.00)		
Missing	0	0	0	0		
Family history of PD					<0.001	0.522
Family members w/PD	103 (24.41%)	19 (21.84%)	84 (25.15%)	10 (5.10%)		
No family members w/PD	319 (75.59%)	68 (78.16%)	250 (74.85%)	186 (94.90%)		
Missing	1	0	1	0		
MDS-UDPRS part I					<0.001	<0.001
Mean (SD)	5.57 (4.07)	7.38 (4.09)	5.10 (3.93)	2.95 (2.96)		
(Min, Max)	(0.00, 24.00)	(1.00, 18.00)	(0.00, 24.00)	(0.00, 17.00)		
Missing	1	0	0	1		
MDS-UDPRS part II					<0.001	0.043
Mean (SD)	5.90 (4.19)	6.71 (4.23)	5.69 (4.16)	0.46 (1.02)		
(Min, Max)	(0.00, 22.00)	(1.00, 18.00)	(0.00, 22.00)	(0.00, 6.00)		
Missing	1	0	0	1		
MDS-UDPRS part III					<0.001	0.105
Mean (SD)	20.89 (8.85)	19.51 (7.65)	21.24 (9.12)	1.21 (2.20)		
(Min, Max)	(4.00, 51.00)	(6.00, 41.00)	(4.00, 51.00)	(0.00, 13.00)		
Missing	0	0	0	2		
H&Y					<0.001	0.620
Stage 0	0 (0.00%)	0 (0.00%)	0 (0.00%)	193 (98.97%)		
Stage 1	185 (43.74%)	42 (48.28%)	143 (42.69%)	2 (1.03%)		
Stage 2	236 (55.79%)	45 (51.72%)	190 (56.72%)	0 (0.00%)		
Stage 3–5	2 (0.47%)	0 (0.00%)	2 (0.60%)	0 (0.00%)		
Missing	0	0	0	1		
PD clinical subtype					N/A	0.484
TD	299 (70.85%)	59 (67.82%)	240 (71.64%)	N/A		
Non-TD	123 (29.85%)	28 (32.18%)	95 (28.36%)	N/A		
Missing	1	0	0	N/A		
Tremor score					<0.001	0.915
Mean (SD)	0.49 (0.32)	0.49 (0.29)	0.49 (0.32)	0.03 (0.08)		
(Min, Max)	(0.00, 1.82)	(0.00, 1.64)	(0.00, 1.82)	(0.00, 0.64)		
Missing	1	0	0	2		
PIGD score					<0.001	0.921
Mean (SD)	0.23 (0.22)	0.22 (0.21)	0.23 (0.23)	0.02 (0.09)		
(Min, Max)	(0.00, 1.40)	(0.00, 1.00)	(0.00, 1.40)	(0.00, 0.08)		
Missing	1	0	0	1		
Side most affected					N/A	0.252
Left	179 (42.32%)	32 (36.78%)	146 (43.58%)	N/A		
Non-Left	244 (57.68%)	55 (63.22%)	189 (56.42%)	N/A		
Missing	0	0	0	N/A		
MoCA					<0.001	0.341
Mean (SD)	27.14 (2.32)	26.89 (2.33)	27.16 (2.31)	28.23 (1.11)		
(Min, Max)	(17.00, 30.00)	(20.00, 30.00)	(17.00, 30.00)	(26.00, 30.00)		
Missing	0	0	0	0		

PD, Parkinson disease; HC, healthy controls; MDS-UPDRS, Movement Disorders Society-Unified Parkinson’s Disease Rating Scale; H&Y, Hoehn and Yahr; PIGD, Postural instability Gait Disorder; TD, tremor dominant; S&E, Modified Schwab and England Activities of Daily Living Scale; MoCA, Montreal Cognitive Assessment.

**Figure 2:**
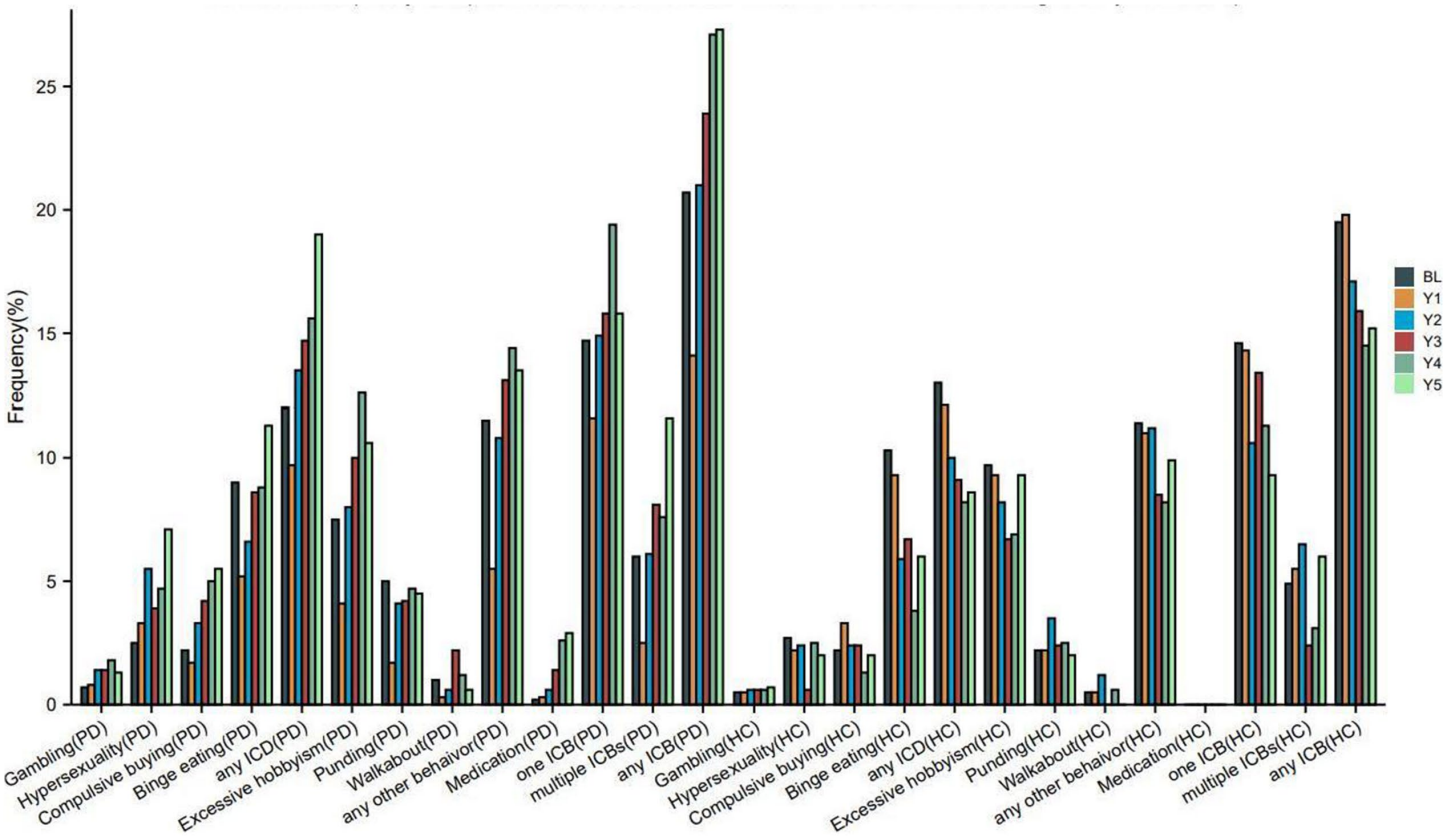
Frequency of impulse control behaviours in PD and HC at baseline and during the 5-years follow up. PD, Parkinson disease; HC, healthy controls; ICBs, Impulse control behaviours; ICD, impulse control disorders; BL, baseline; Y1, year 1; Y2, year 2; Y3, year 3; Y4, year 4; Y5, year 5. Each instance of ICB consists of a single ICB as well as cases where it coexists with other ICBs. For example, as long as that patient has gambling that patient is in the Gambling group, whether or not that patient combines other ICBs.

Figure 2 and Table 2 illustrate the longitudinal changes in the occurrence of ICBs among PD and HC participants. The results show a significant increase in the proportion of PD participants classified as having ICBs over time (*p* < 0.001). In contrast, HC participants demonstrated a significant decrease in the proportion classified as having ICBs (*p* = 0.005). Furthermore, there was a significant difference in the rates of change in ICBs over time between the PD and HC groups (group × visit interaction, *p* = 0.001).

**Table 2:** Impulse control behaviours over time in PD and HC.

Variable	Patients with PD							HC							*p* Values	
	BL *n* = 401	Year 1 *n* = 362	Year 2 *n* = 362	Year 3 *n* = 360	Year 4 *n* = 340	Year 5 *n* = 311	*p* Value (change over time)	BL *n* = 185	Year 1 *n* = 182	Year 2 *n* = 170	Year 3 *n* = 164	Year 4 *n* = 159	Year 5 *n* = 151	*p* Value (change over time)	Group × visit interaction	PD *vs* HC
ICBs							<0.001							0.005	<0.001	N/A^a^
Positive	83 (20.7%)	51 (14.1%)	76 (21.0%)	86 (23.9%)	92 (27.1%)	85 (27.3%)		36 (19.5%)	36 (19.8%)	29 (17.1%)	26 (15.9%)	23 (14.5%)	23 (15.2%)			
Negative	318 (79.3%)	311 (85.9%)	286 (79.0%)	274 (76.1%)	248 (72.9%)	226 (72.7%)		149 (80.5%)	146 (80.2%)	141 (82.9%)	138 (84.1%)	136 (85.5%)	128 (84.8%)			
ICDs							<0.001							<0.001	<0.001	N/A^a^
Positive	48 (12.0%)	35 (9.7%)	49 (13.5%)	53 (14.7%)	53 (15.6%)	59 (19.0%)		24 (13.0%)	22 (12.1%)	17 (10.0%)	15 (9.1%)	13 (8.2%)	13 (8.6%)			
Negative	353 (88.0%)	327 (90.3%)	313 (86.5%)	307 (85.3%)	287 (84.4%)	252 (81.0%)		161 (87.0%)	160 (87.9%)	153 (90.0%)	149 (90.9%)	146 (91.8%)	138 (91.4%)			
Other behaivors							<0.001							0.014	<0.001	N/A^a^
Positive	46 (11.5%)	20 (5.5%)	39 (10.8%)	47 (13.1%)	49 (14.4%)	42 (13.5%)		21 (11.4%)	20 (11.0%)	19 (11.2%)	14 (8.5%)	13 (8.2%)	15 (9.9%)			
Negative	355 (88.5%)	342 (94.5%)	323 (89.2%)	313 (86.9%)	291 (85.6%)	269 (86.5%)		164 (88.6%)	162 (89.0%)	151 (88.8%)	150 (91.5%)	146 (91.8%)	136 (90.1%)			
DDS							<0.001							NA^b^	NA^b^	NA^b^
Positive	1 (0.2%)	1 (0.3%)	2 (0.6%)	5 (1.4%)	9 (2.6%)	9 (2.9%)		0 (0.0%)	0 (0.0%)	0 (0.0%)	0 (0.0%)	0 (0.0%)	0 (0.0%)			
Negative	400 (99.8%)	361 (99.7%)	360 (99.4%)	355 (98.6%)	331 (97.4%)	302 (97.1%)		185 (100.0%)	182 (100.0%)	170 (100.0%)	164 (100.0%)	159 (100.0%)	151 (100.0%)			
Pathological gambling							<0.001							0.861	<0.001	N/A^a^
Positive	3 (0.7%)	3 (0.8%)	5 (1.4%)	5 (1.4%)	6 (1.8%)	4 (1.3%)		1 (0.5%)	1 (0.5%)	1 (0.6%)	1 (0.6%)	1 (0.6%)	1 (0.7%)			
Negative	398 (99.3%)	359 (99.2%)	357 (98.6%)	355 (98.6%)	334 (98.2%)	307 (98.7%)		184 (99.5%)	181 (99.5%)	169 (99.4%)	163 (99.4%)	158 (99.4%)	150 (99.3%)			
Hypersexuality							<0.001							0.003	<0.001	N/A^a^
Positive	10 (2.5%)	12 (3.3%)	20 (5.5%)	14 (3.9%)	16 (4.7%)	22 (7.1%)		5 (2.7%)	4 (2.2%)	4 (2.4%)	1 (0.6%)	4 (2.5%)	3 (2.0%)			
Negative	391 (97.5%)	350 (96.7%)	342 (94.5%)	346 (96.1%)	324 (95.3%)	289 (92.9%)		180 (97.3%)	178 (97.8%)	166 (97.6%)	163 (99.4%)	155 (97.5%)	148 (98.0%)			
Compulsive buying							<0.001							0.012	<0.001	N/A^a^
Positive	9 (2.2%)	6 (1.7%)	12 (3.3%)	15 (4.2%)	17 (5.0%)	17 (5.5%)		4 (2.2%)	6 (3.3%)	4 (2.4%)	4 (2.4%)	2 (1.3%)	3 (2.0%)			
Negative	392 (97.8%)	356 (98.3%)	350 (96.7%)	345 (95.8%)	323 (95.0%)	294 (94.5%)		181 (97.8%)	176 (96.7%)	166 (97.6%)	160 (97.6%)	157 (98.7%)	148 (98.0%)			
Binge eating							<0.001							<0.001	<0.001	N/A^a^
Positive	36 (9.0%)	19 (5.2%)	24 (6.6%)	31 (8.6%)	30 (8.8%)	35 (11.3%)		19 (10.3%)	17 (9.3%)	10 (5.9%)	11 (6.7%)	6 (3.8%)	9 (6.0%)			
Negative	365 (91.0%)	343 (94.8%)	338 (93.4%)	329 (91.4%)	310 (91.2%)	276 (88.7%)		166 (89.7%)	165 (90.7%)	160 (94.1%)	153 (93.3%)	153 (96.2%)	142 (94.0%)			
Excessive hobbyism							<0.001							0.038	<0.001	N/A^a^
Positive	30 (7.5%)	15 (4.1%)	29 (8.0%)	36 (10.0%)	43 (12.6%)	33 (10.6%)		18 (9.7%)	17 (9.3%)	14 (8.2%)	11 (6.7%)	11 (6.9%)	14 (9.3%)			
Negative	371 (92.5%)	347 (95.9%)	333 (92.0%)	324 (90.0%)	297 (87.4%)	278 (89.4%)		167 (90.3%)	165 (90.7%)	156 (91.8%)	153 (93.3%)	148 (93.1%)	137 (90.7%)			
Punding							0.069							0.073	0.030	N/A^a^
Positive	20 (5.0%)	6 (1.7%)	15 (4.1%)	15 (4.2%)	16 (4.7%)	14 (4.5%)		4(2.2%)	4(2.2%)	6(3.5%)	4(2.4%)	4(2.5%)	3(2.0%)			
Negative	381 (95.0%)	356 (98.3%)	347 (95.9%)	345 (95.8%)	324 (95.3%)	297 (95.5%)		181(97.8%)	178(97.8%)	164(96.5%)	160(97.6%)	155(97.5%)	148(98.0%)			
Walkabout							0.595							1.000	0.400	0.212
Positive	4 (1.0%)	1 (0.3%)	2 (0.6%)	8 (2.2%)	4 (1.2%)	2 (0.6%)		1 (0.5%)	1 (0.5%)	2 (1.2%)	0 (0.0%)	1 (0.6%)	0 (0.0%)			
Negative	397 (99.0%)	361 (99.7%)	360 (99.4%)	352 (97.8%)	336 (98.8%)	309 (99.4%)		184 (99.5%)	181 (99.5%)	168 (98.8%)	164 (100.0%)	158 (99.4%)	151 (100.0%)			
Clinical subtypes																
One ICB	59 (14.7%)	42 (11.6%)	54 (14.9%)	57 (15.8%)	66 (19.4%)	49 (15.8%)	0.033	27 (14.6%)	26 (14.3%)	18 (10.6%)	22 (13.4%)	18 (11.3%)	14 (9.3%)	0.019	0.003	N/A^a^
Multiple ICBs	24 (6.0%)	9 (2.5%)	22 (6.1%)	29 (8.1%)	26 (7.6%)	36 (11.6%)	<0.001	9 (4.9%)	10 (5.5%)	11 (6.5%)	4 (2.4%)	5 (3.1%)	9 (6.0%)	0.024	<0.001	N/A^a^
Negative	318 (79.3%)	311 (85.9%)	286 (79.0%)	274 (76.1%)	248 (72.9%)	226 (72.7%)		149 (80.5%)	146 (80.2%)	141 (82.9%)	138 (84.1%)	136 (85.5%)	128 (84.8%)			

PD, Parkinson disease; HC, healthy controls; ICBs, Impulse control behaviours; ICD, impulse control disorders; DDS, dopamine dysregulation syndrome.

a PD versus HC comparison is not applicable if test of interaction was significant.

b There were not enough positive results in the HC group to run analysis.

Furthermore, the proportion of PD participants with ICDs (*p* < 0.001), any other behavior (*p* < 0.001), or DDS (*p* < 0.001) also showed a significant increase over time. On the other hand, HC participants with ICDs (*p* < 0.001) or any other behavior (*p* = 0.014) demonstrated a significant longitudinal decrease in the proportion (Table 2). However, there were too few HC participants with DDS to perform a longitudinal analysis on this group. Additionally, the rates of change in ICDs or any other behavior over time differed significantly between the PD and HC groups.

Regarding specific symptoms, the proportion of PD participants with PG (*p* < 0.001), HS (*p* < 0.001), CB (*p* < 0.001), BE (*p* < 0.001), or excessive hobbyism (*p* < 0.001) also significantly increased over time. Conversely, HC participants with HS (*p* = 0.003), CB (*p* = 0.012), BE (*p* < 0.001), or excessive hobbyism (*p* = 0.038) demonstrated a significant longitudinal decrease in the proportion (Table 2). Additionally, the rates of change in PG, HS, CB, BE, excessive hobbyism, or punding over time differed significantly between the PD and HC groups.

Regarding clinical subtypes, the proportion of PD participants with one ICB (*p* = 0.033) or multiple ICBs (*p* < 0.001) significantly increased over time. Conversely, HC participants with one ICB (*p* = 0.019) or multiple ICBs (*p* = 0.024) demonstrated a significant longitudinal decrease in the proportion (Table 2). Additionally, the rates of change in the percentage of one ICB or multiple ICBs over time differed significantly between the PD and HC groups.

### 3.2. Longitudinal assessment of impulse control behaviours in PD

The demographic characteristics and PD characteristics of participants with PD who had ICBs at baseline (*n* = 83) and those without ICBs (*n* = 318) are presented in Table 1. There were no significant differences in terms of demographics between the PD participants with and without ICBs.

The motor and non-motor characteristics of participants with PD with and without ICBs over the 5-year follow-up period are presented in Table 3. At each time point, subjects with ICBs had higher scores on part I of the MDS-UPDRS, which assesses neuropsychiatric and non-motor symptoms. They were also more likely to have worse scores on part II, which evaluates patient-completed experiences of daily living. However, there were no significant differences in part III of the MDS-UPDRS, which measures motor symptoms, or in the Hoehn and Yahr stage between the two groups.

**Table 3:** PD motor and non-motor characteristics over time by ICBs status.

Variable	Baseline			Year 1			Year 2			Year 3			Year 4			Year 5		
	PD ICBs+ *n* = 83	PD ICBs− *n* = 318	*p* Value	PD ICBs+ *n* = 51	PD ICBs− *n* = 311	*p* Value	PD ICBs+ *n* = 76	PD ICBs− *n* = 286	*p* Value	PD ICBs+ *n* = 86	PD ICBs− *n* = 274	*p* Value	PD ICBs+ *n* = 92	PD ICBs− *n* = 248	*p* Value	PD ICBs+ *n* = 85	PD ICBs− *n* = 226	*p* Value
MDS-UDPRS part I			<0.001			0.014			0.006			0.001			0.004			0.001
Mean (SD)	7.16 (3.93)	5.03 (3.94)		8.29 (5.33)	6.56 (4.54)		9.07 (4.69)	7.29 (5.09)		10.35 (6.96)	7.68 (4.72)		10.50 (6.29)	8.49 (5.49)		11.34 (6.59)	8.47 (5.62)	
(Min, Max)	(1.00, 18.00)	(0.00, 24.00)		(0.00, 29.00)	(0.00, 27.00)		(0.00, 26.00)	(0.00, 25.00)		(0.00, 36.00)	(0.00, 25.00)		(0.00, 29.00)	(0.00, 30.00)		(1.00, 34.00)	(0.00, 31.00)	
Missing	0	0		0	0		0	0		0	0		0	0		0	0	
MDS-UDPRS part II			0.032			0.021			0.104			0.011			0.020			0.004
Mean (SD)	6.70 (4.27)	5.60 (4.12)		8.86 (5.81)	7.14 (4.74)		8.91 (5.58)	7.79 (5.20)		10.24 (5.86)	8.47 (5.55)		11.15 (7.40)	9.29 (6.14)		11.76 (6.38)	9.36 (6.54)	
(Min, Max)	(1.00, 18.00)	(0.00, 22.00)		(0.00, 30.00)	(0.00, 25.00)		(0.00, 23.00)	(0.00, 27.00)		(0.00, 29.00)	(0.00, 29.00)		(1.00, 37.00)	(0.00, 35.00)		(1.00, 31.00)	(0.00, 40.00)	
Missing	0	0		0	1		1	1		0	0		1	0		0	0	
MDS-UDPRS part III			0.197			0.444			0.792			0.841			0.709			0.820
Mean (SD)	19.70 (7.73)	21.11 (9.10)		25.53 (12.13)	24.12 (9.97)		27.41 (13.28)	26.96 (10.84)		28.74 (11.46)	28.43 (12.37)		31.11 (12.58)	30.51 (12.38)		30.10 (12.49)	30.49 (13.77)	
(Min, Max)	(6.00, 41.00)	(4.00, 51.00)		(4.00, 56.00)	(2.00, 60.00)		(6.00, 68.00)	(3.00, 59.00)		(4.00, 57.00)	(4.00, 80.00)		(6.00, 63.00)	(6.00, 80.00)		(7.00, 66.00)	(3.00, 90.00)	
Missing	0	0		2	24		6	29		5	29		8	27		1	16	
MDS-UDPRS part IV			NA			0.029			0.423			0.516			0.577			0.282
Mean (SD)	NA	NA		0.94 (1.69)	0.25 (0.97)		0.52 (1.34)	0.72 (1.85)		0.87 (1.77)	1.04 (2.00)		1.42 (2.54)	1.61 (2.66)		1.83 (3.02)	2.24 (2.93)	
(Min, Max)				(0.00, 5.00)	(0.00, 7.00)		(0.00, 6.00)	(0.00, 11.00)		(0.00, 6.00)	(0.00, 11.00)		(0.00, 13.00)	(0.00, 12.00)		(0.00, 16.00)	(0.00, 17.00)	
Missing				18	127		14	42		9	19		7	10		2	12	
H&Y			0.762			0.874			0.169			0.660			0.888			0.969
Stage 0	0 (0.00%)	0 (0.00%)		0 (0.00%)	1 (0.35%)		1 (1.43%)	0 (0.00%)		0 (0.00%)	1 (0.41%)		0 (0.00%)	1 (0.45%)		0 (0.00%)	0 (0.00%)	
Stage 1	39 (46.99%)	138 (43.40%)		14 (28.57%)	83 (28.72%)		13 (18.57%)	65 (25.19%)		13 (16.05%)	48 (19.59%)		13 (15.29%)	32 (14.48%)		9 (10.72%)	22 (10.38%)	
Stage 2	44 (53.01%)	178 (55.97%)		34 (69.39%)	193 (66.78%)		51 (72.86%)	181 (70.16%)		64 (79.01%)	177 (72.24%)		64 (75.29%)	163 (73.76%)		68 (80.95%)	174 (82.07%)	
Stage 3–5	0 (0.00%)	2 (0.63%)		1 (2.04%)	12 (4.15%)		5 (7.14%)	12 (4.65%)		4 (4.94%)	19 (7.76%)		8(9.41%)	25 (11.31%)		7 (8.33%)	16 (7.55%)	
Missing	0	0		2	22		6	28		5	29		7	27		1	14	
PD clinical subtype			0.523			0.328			0.062			0.132			0.334			0.207
TD	56 (67.47%)	226 (71.07%)		35 (71.43%)	185 (64.24%)		39 (55.71%)	174 (67.70%)		42 (55.56%)	159 (64.90%)		54 (63.53%)	127 (57.47%)		42 (50.00%)	122 (58.10%)	
non-TD	27 (32.53%)	92 (28.93%)		14 (28.57%)	103 (35.76%)		31 (44.29%)	83 (32.30%)		36 (45.44%)	86 (35.10%)		31 (36.47%)	94 (42.53%)		42 (50.00%)	88 (41.90%)	
Missing	0	0		2	23		6	29		5	29		7	27		1	16	
Tremor score			0.981			0.043			0.611			0.418			0.540			0.449
Mean (SD)	0.49 (0.29)	0.49 (0.32)		0.65 (0.43)	0.53 (0.37)		0.56 (0.46)	0.59 (0.42)		0.57 (0.42)	0.62 (0.45)		0.68 (0.48)	0.65 (0.47)		0.59 (0.45)	0.63 (0.46)	
(Min, Max)	(0.00, 1.64)	(0.00, 1.82)		(0.00, 2.00)	(0.00, 1.55)		(0.00, 2.18)	(0.00, 2.45)		(0.00, 1.82)	(0.00, 2.27)		(0.00, 1.73)	(0.00, 2.09)		(0.00, 1.82)	(0.00, 2.09)	
Missing	0	0		2	20		6	28		5	29		7	27		1	14	
PIGD score			0.985			0.781			0.220			0.551			0.726			0.357
Mean (SD)	0.23 (0.22)	0.23 (0.23)		0.32 (0.25)	0.31 (0.31)		0.40 (0.46)	0.34 (0.36)		0.43 (0.36)	0.40 (0.45)		0.47 (0.41)	0.49 (0.52)		0.51 (0.49)	0.52 (0.56)	
(Min, Max)	(0.00, 1.00)	(0.00, 1.40)		(0.00, 1.00)	(0.00, 1.80)		(0.00, 3.00)	(0.00, 2.60)		(0.00, 2.40)	(0.00, 3.00)		(0.00, 1.80)	(0.00, 3.40)		(0.00, 4.00)	(0.00, 3.60)	
Missing	0	0		2	23		6	29		5	29		7	27		1	14	
Side most affected			0.488			0.021			0.148			0.308			0.179			0.243
Left	32 (38.55%)	136 (42.77%)		14 (27.45%)	139 (44.69%)		27 (35.53%)	128 (44.76%)		32 (37.21%)	119 (43.43%)		33 (35.87%)	109 (43.95%)		31 (36.47%)	99 (43.81%)	
non-Left	51 (61.45%)	182 (57.23%)		37 (72.55%)	172 (55.31%)		49 (64.47%)	158 (55.24%)		54 (62.79%)	155 (56.57%)		59 (64.13%)	139 (56.05%)		54 (63.53%)	127 (56.19%)	
Missing	0	0		0	0		0	0		0	0		0	0		0	0	
S&E			0.557			0.700			0.150			0.821			0.480			0.141
Mean (SD)	93.55 (5.50)	93.13 (5.96)		91.00 (5.35)	90.61 (6.81)		87.53 (6.70)	89.04 (8.35)		87.53 (8.08)	87.76 (8.02)		86.41 (9.03)	85.57 (10.03)		83.35 (11.94)	85.51 (11.29)	
(Min, Max)	(70.00, 100.00)	(75.00, 100.00)		(80.00, 100.00)	(70.00, 100.00)		(70.00,100.00)	(60.00, 100.00)		(50.00, 100.00)	(60.00, 100.00)		(50.00, 100.00)	(30.00, 100.00)		(20.00, 100.00)	(20.00, 100.00)	
Missing	0	0		1	0		1	0		1	0		0	2		0	0	
MoCA			0.341			0.804			0.785			0.981			0.980			0.337
Mean (SD)	26.89 (2.33)	27.16 (2.31)		26.47 (2.66)	26.31 (2.84)		26.13 (3.05)	26.24 (3.19)		26.36 (3.20)	26.36 (3.07)		26.45 (3.78)	26.46 (3.39)		26.89 (2.79)	26.47 (3.69)	
(Min, Max)	(20.00, 30.00)	(17.00, 30.00)		(20.00, 30.00)	(15.00, 30.00)		(16.00, 30.00)	(9.00, 30.00)		(15.00, 30.00)	(13.00, 30.00)		(11.00, 30.00)	(11.00, 30.00)		(17.00, 30.00)	(2.00, 30.00)	
Missing	0	0		0	1		0	2		1	1		1	3		1	1	
GDS			<0.001			0.010			0.004			0.001			0.003			<0.001
Mean (SD)	3.23 (2.53)	2.08 (2.40)		3.55 (3.20)	2.42 (2.85)		3.53 (2.98)	2.44 (2.84)		3.49 (2.97)	2.36 (2.75)		3.47 (3.25)	2.32 (2.61)		3.74 (3.02)	2.39 (2.52)	
(Min, Max)	(0.00, 11.00)	(0.00, 14.00)		(0.00, 15.00)	(0.00, 14.00)		(0.00, 14.00)	(0.00, 15.00)		(0.00, 13.00)	(0.00, 14.00)		(0.00, 15.00)	(0.00, 15.00)		(0.00, 11.00)	(0.00, 13.00)	
Missing	0	0		0	1		0	2		0	0		0	0		0	0	
STAI—state subscore			0.005			0.429			0.005			0.016			0.002			<0.001
Mean (SD)	35.77 (10.25)	32.20 (10.17)		33.48 (9.07)	32.28 (10.06)		35.54 (9.61)	31.87 (10.16)		34.38 (10.05)	31.41 (9.83)		34.90 (10.49)	31.21 (9.49)		35.88 (10.49)	30.55 (9.26)	
(Min, Max)	(20.00, 64.00)	(20.00, 76.00)		(20.00, 53.00)	(20.00, 69.00)		(20.00, 60.00)	(20.00, 76.00)		(20.00, 68.00)	(20.00, 71.00)		(20.00, 63.00)	(20.00, 73.00)		(20.00, 70.00)	(20.00, 75.00)	
Missing	0	2		1	0		0	2		0	3		0	1		0	1	
STAI—trait subscore			<0.001			0.004			<0.001			0.001			<0.001			<0.001
Mean (SD)	36.96 (8.91)	31.18 (9.27)		36.34 (9.81)	32.18 (9.44)		36.29 (9.35)	31.69 (9.44)		35.88 (9.31)	31.82 (9.81)		36.89 (10.49)	31.21 (9.28)		37.82 (11.30)	30.78 (8.99)	
(Min, Max)	(23.00, 55.00)	(20.00, 63.00)		(21.00, 58.00)	(20.00, 73.00)		(21.00, 66.00)	(20.00, 66.00)		(22.00, 59.00)	(20.00, 64.00)		(20.00, 62.00)	(20.00, 69.00)		(20.00, 68.00)	(20.00, 75.00)	
Missing	0	1		1	1		1	5		0	3		0	4		0	1	
RBD			0.104			0.091			0.340			0.798			0.458			0.207
Positive (≥5)	36 (44.44%)	110 (34.70%)		11 (21.57%)	104 (33.44%)		32 (42.11%)	103 (36.14%)		36 (41.86%)	119 (43.43%)		44 (47.82%)	107 (43.32%)		34 (40.00%)	108 (48.00%)	
Negative (<5)	45 (55.56%)	207 (65.30%)		40 (78.43%)	207 (66.56%)		44 (57.89%)	182 (63.86%)		50 (58.14%)	155 (56.57%)		48 (52.18%)	140 (56.68%)		51 (60.00%)	117 (52.00%)	
Missing	2	1		0	0		0	0		0	0		0	1		0	1	
ESS			0.057			0.039			0.007			<0.001			0.002			<0.001
Mean (SD)	6.32 (3.31)	5.52 (3.37)		7.27 (4.69)	6.03 (3.87)		7.87 (4.23)	6.42 (4.12)		9.06 (5.12)	6.80 (4.16)		8.75 (5.11)	6.96 (4.41)		9.58 (5.08)	7.06 (4.40)	
(Min, Max)	(1.00, 15.00)	(0.00, 20.00)		(0.00, 21.00)	(0.00, 18.00)		(0.00, 22.00)	(0.00, 23.00)		(2.00, 24.00)	(0.00, 19.00)		(0.00, 24.00)	(0.00, 22.00)		(1.00, 24.00)	(0.00, 24.00)	
Missing	1	0		0	0		0	1		0	3		0	0		0	0	
SCOPA-AUT			<0.001			0.026			<0.001			<0.001			0.003			<0.001
Mean (SD)	12.51 (7.10)	8.53 (5.43)		12.86 (7.95)	10.65 (6.20)		13.96 (7.48)	10.83 (6.13)		15.40 (7.36)	11.50 (6.73)		15.07 (8.65)	12.01 (6.89)		16.92 (9.91)	12.31 (6.86)	
(Min, Max)	(2.00, 39.00)	(0.00, 32.00)		(0.00, 45.00)	(0.00, 39.00)		(3.00, 42.00)	(0.00, 37.00)		(2.00, 30.00)	(0.00, 34.00)		(2.00, 42.00)	(0.00, 39.00)		(1.00, 45.00)	(0.00, 41.00)	
Missing	0	6		1	4		0	3		1	2		0	0		0	0	

PD, Parkinson disease; ICBs, Impulse control behaviours; MDS-UPDRS, Movement Disorders Society-Unified Parkinson’s Disease Rating Scale; H&Y, Hoehn and Yahr; PIGD, Postural instability Gait Disorder; TD, tremor dominant; S&E, Modified Schwab and England Activities of Daily Living Scale; MoCA, Montreal Cognitive Assessment; GDS, Geriatric Depression Scale; STAI, State–Trait Anxiety Inventory; ESS, Epworth Sleepiness Scale; RBD, rapid eye movement sleep behaviour disorder; SCOPA-AUT, Scales for Outcomes in Parkinson’s Disease-Autonomic; NA, not applicable.

At year 1 only, subjects with ICBs had worse scores on part IV of the MDS-UPDRS, which assesses motor complications. They also had higher tremor scores and were more likely to be affected on the left side. There were no significant differences in clinical subtype or PIGD scores between the two groups.

Regarding the association of ICBs with other NMS, there were no significant differences in activities of daily living, cognition, or RBD between the groups at any time point. However, similar to the baseline findings in this cohort, ICBs were associated with depression, autonomic dysfunction, and anxiety at each time point (Table 3). Although there was no statistical difference in the ESS at baseline, subjects with ICBs had worse ESS scores over the next 5 years (Table 3).

Regarding dopaminergic therapy, subjects with ICBs had higher total LEDD at year 3 only, but there were no significant differences in the LEDD subtotal for dopamine agonists or non-dopamine agonists between the groups at any time point (Table 4). Regarding the use of specific drug types, subjects with ICBs had a higher frequency of Dopamine agonist use at year 3 and 4, as well as a higher frequency of MAO-B inhibitor use at year 3. However, there were no significant differences in the use of Levodopa, entacapone, amantadine, or anticholinergic drugs between the groups at any time point (Table 4).

**Table 4:** Medication use over time by ICBs status.

Variable	Treated at Year 1			Treated at Year 2			Treated at Year 3			Treated at Year 4			Treated at Year 5		
	PD ICBs+ *n* = 51	PD ICBs− *n* = 311	*p* Value	PD ICBs+ *n* = 76	PD ICBs− *n* = 286	*p* Value	PD ICBs+ *n* = 86	PD ICBs− *n* = 274	*p* Value	PD ICBs+ *n* = 92	PD ICBs− *n* = 248	*p* Value	PD ICBs+ *n* = 85	PD ICBs− *n* = 226	*p* Value
Total LEDD			0.480			0.803			0.040			0.899			0.067
Mean (SD)	177.39 (185.24)	203.48 (252.30)		357.42 (265.83)	347.15 (330.90)		563.22 (629.12)	439.30 (433.89)		539.56 (293.67)	532.94 (467.45)		792.68 (1139.88)	622.97 (487.83)	
(Min, Max)	(0.00, 750.00)	(0.00, 1740.00)		(0.00, 1140.00)	(0.00, 2314.20)		(0.00, 5300.00)	(0.00, 5000.00)		(0.00, 1670.00)	(0.00, 5360.00)		(0.00, 10300.00)	(0.00, 5460.00)	
Missing	0	0		0	0		0	0		0	0		0	0	
LEDD subtotal—dopamine agonists			0.348			0.578			0.133			0.726			0.644
Mean (SD)	56.81 (107.55)	41.92 (80.94)		66.43 (86.95)	76.64 (153.40)		129.69 (269.70)	87.83 (209.24)		95.27 (114.06)	104.90 (253.33)		104.52 (176.04)	93.53 (187.17)	
(Min, Max)	(0.00, 450.00)	(0.00, 391.80)		(0.00, 320.00)	(0.00, 1638.00)		(0.00, 2058.00)	(0.00, 2880.00)		(0.00, 450.00)	(0.00, 2880.00)		(0.00, 1460.40)	(0.00, 1396.50)	
Missing	0	0		0	0		0	0		0	0		0	0	
LEDD subtotal—non-dopamine agonists			0.104			0.603			0.152			0.738			0.086
Mean (SD)	120.59 (148.38)	161.56 (244.33)		290.99 (255.30)	270.51 (316.87)		433.53 (600.81)	351.47 (410.54)		444.29 (296.16)	428.05 (427.70)		688.16 (1142.50)	529.44 (481.01)	
(Min, Max)	(0.00, 600.00)	(0.00, 1740.00)		(0.00, 1140.00)	(0.00, 2314.20)		(0.00, 5300.00)	(0.00, 5000.00)		(0.00, 1670.00)	(0.00, 5360.00)		(0.00, 10300.00)	(0.00, 5300.00)	
Missing	0	0		0	0		0	0		0	0		0	0	
classes of PD medications															
Levodopa	14 (27.45%)	82 (26.37%)	0.871	40 (52.63%)	128 (44.76%)	0.221	55 (63.95%)	169 (61.68%)	0.704	71 (77.17%)	170 (68.55%)	0.120	74 (87.06%)	1764 (77.88%)	0.069
Entacapone	0 (0.00%)	0 (0.00%)	NA^a^	0 (0.00%)	3 (1.05%)	NA^a^	3 (3.49%)	5 (1.828%)	0.403	2 (2.17%)	11 (4.44%)	0.526	5 (5.88%)	10 (4.42%)	0.564
MAO-B inhibitors	12 (23.53%)	96 (30.87%)	0.288	37 (48.68%)	108 (37.76%)	0.084	45 (52.33%)	107 (39.05%)	0.030	41 (44.57%)	102 (41.13%)	0.569	40 (47.06%)	92 (40.70%)	0.313
Dopamine agonists	16 (31.37%)	84 (27.01%)	0.518	34 (44.73%)	104 (36.36%)	0.182	45 (52.33%)	107 (39.05%)	0.030	51 (55.43%)	98 (39.52%)	0.009	42 (49.41%)	88 (38.94%)	0.095
Amantadine	6 (11.76%)	21 (6.75%)	0.245	9 (11.84%)	34(11.89%)	0.991	18 (20.93%)	35 (12.77%)	0.063	12 (13.04%)	41 (16.53%)	0.431	13 (15.29%)	39 (17.26%)	0.679
Anticholinergics	1 (1.96%)	4 (1.27%)	0.534	0 (0.00%)	6(2.10%)	0.350	3 (3.49%)	8 (2.92%)	0.728	1 (1.09%)	7 (2.82%)	0.688	3 (3.53%)	7 (3.10%)	1.000

PD, Parkinson disease; ICBs, Impulse control behaviours; LEDD, levodopa equivalent daily dose; MAO-B, Monoamine oxidase-B.

a There were not enough positive results to run analysis.

Regarding presynaptic dopaminergic dysfunction as measured by DaT scan in relation to clinical symptoms, there were no significant differences in terms of contralateral caudate, ipsilateral caudate, contralateral putamen, or ipsilateral putamen at baseline, year 1, 2, and 4 (Table 5). However, data for year 3 and 5 were not yet available at the time of data access.

**Table 5:** Presynaptic dopaminergic dysfunction over time as measured by DaTscan.

Variable	Baseline			Year 1			Year 2			Year 3			Year 4		
	PD ICBs+ *n* = 83	PD ICBs− *n* = 318	*p* Value	PD ICBs+ *n* = 51	PD ICBs− *n* = 311	*p* Value	PD ICBs+ *n* = 76	PD ICBs− *n* = 286	*p* Value	PD ICBs+ *n* = 86	PD ICBs− *n* = 274	*p* Value	PD ICBs+ *n* = 92	PD ICBs− *n* = 248	*p* Value
Contralateral caudate			0.685			0.986			0.741			NA			0.223
Mean (SD)	1.83 (0.63)	1.81 (0.51)		1.64 (0.49)	1.64 (0.50)		1.51 (0.56)	1.53 (0.51)		NA	NA		1.29 (0.43)	1.37 (0.52)	
(Min, Max)	(0.57, 3.70)	(0.35, 3.57)		(0.63, 3.01)	(0.26, 3.58)		(0.06, 3.02)	(0.48, 3.52)					(0.20, 2.61)	(0.13, 3.09)	
Missing	5	12		4	18		10	18					12	38	
Ipsilateral caudate			0.183			0.227			0.666			NA			0.679
Mean (SD)	2.21 (0.66)	2.11 (0.56)		2.02 (0.62)	1.92 (0.54)		1.84 (0.64)	1.80 (0.56)		NA	NA		1.59 (0.55)	1.62 (0.55)	
(Min, Max)	(0.68, 3.75)	(0.42, 3.98)		(0.60, 3.25)	(0.31, 3.81)		(0.27, 3.48)	(0.25, 3.72)					(0.40, 3.24)	(0.33, 3.75)	
Missing	5	12		4	18		10	18					12	38	
Contralateral putamen			0.112			0.610			0.803			NA			0.172
Mean (SD)	0.72 (0.27)	0.67 (0.25)		0.59 (0.17)	0.61 (0.24)		0.56 (0.22)	0.56 (0.21)		NA	NA		0.47 (0.19)	0.51 (0.21)	
(Min, Max)	(0.27, 2.16)	(0.12, 1.74)		(0.03, 1.01)	(0.07, 1.93)		(0.07, 1.36)	(0.03, 1.52)					(0.07, 0.99)	(0.05, 1.61)	
Missing	5	12		4	18		10	18					12	38	
Ipsilateral putamen			0.767			0.579			0.849			NA			0.874
Mean (SD)	0.94 (0.39)	0.95 (0.37)		0.82 (0.36)	0.79 (0.32)		0.72 (0.34)	0.73 (0.30)		NA	NA		0.61 (0.25)	0.61 (0.25)	
(Min, Max)	(0.25, 2.18)	(0.22, 2.60)		(0.24, 2.21)	(0.03, 2.70)		(0.01, 2.12)	(0.07, 1.91)					(0.16, 1.73)	(0.01, 1.60)	
Missing	5	12		4	18		10	18					12	38	

DaTscan data of year 3 and year 5 are not yet available. PD, Parkinson disease; ICBs, Impulse control behaviours; DaT scan, dopamine transporter deficit on 123I ioflupane imaging.

Regarding biological data, there were also no significant differences between groups in CSF biomarkers, serum urate, or neurofilament light (NfL) at baseline and year 1 (Table 6). However, data for year 2–5 were not yet available at the time of data access.

**Table 6:** Biologics at baseline and year 1 in participants with PD by ICBs status.

Variable	Baseline			Year 1		
	PD ICBs+ *n* = 83	PD ICBs− *n* = 318	*p* Value	PD ICBs+ *n* = 51	PD ICBs− *n* = 311	*p* Value
A-beta (pg/mL)			0.787			0.708
Mean (SD)	369.24 (105.61)	372.10 (100.29)		370.53 (106.44)	377.51 (104.11)	
(Min, Max)	(155.60, 669.20)	(129.20, 796.50)		(184.40, 578.90)	(144.10, 732.50)	
Missing	3	8		26	172	
T-tau (pg/mL)			0.435			0.823
Mean (SD)	42.86 (17.60)	44.67 (17.99)		41.98 (12.80)	42.97 (18.42)	
(Min, Max)	(15.60, 99.30)	(14.40, 121.00)		(25.40, 70.10)	(16.60, 128.80)	
Missing	3	12		26	173	
p-tau (pg/mL)			0.764			0.446
Mean (SD)	16.07 (10.62)	15.56 (9.99)		17.36 (11.76)	18.66 (11.88)	
(Min, Max)	(4.70, 67.00)	(5.70, 94.10)		(6.60, 47.50)	(5.40, 61.80)	
Missing	3	10		26	173	
Alpha-synuclein (pg/mL)			0.999			0.509
Mean (SD)	1826.88 (765.21)	1857.83 (802.35)		1741.05 (621.56)	1889.30 (827.64)	
(Min, Max)	(363.12, 4709.78)	(332.93, 6694.55)		(797.87, 3438.47)	(352.36, 5157.08)	
Missing	3	8		26	172	
T-tau/A-beta			0.316			0.402
Mean (SD)	0.12 (0.06)	0.13 (0.06)		0.12 (0.06)	0.12 (0.07)	
(Min, Max)	(0.06, 0.49)	(0.04, 0.52)		(0.08, 0.36)	(0.06, 0.51)	
Missing	3	12		26	173	
p-tau/A-beta			0.781			0.619
Mean (SD)	0.05 (0.03)	0.04 (0.04)		0.05 (0.05)	0.05 (0.04)	
(Min, Max)	(0.02, 0.26)	(0.01, 0.51)		(0.02, 0.26)	(0.01, 0.28)	
Missing	3	10		26	173	
p-tau/T-tau			0.301			0.570
Mean (SD)	0.39 (0.21)	0.37 (0.23)		0.41 (0.22)	0.48 (0.36)	
(Min, Max)	(0.12, 1.04)	(0.14, 2.14)		(0.18, 1.00)	(0.07, 2.48)	
Missing	3	14		26	174	
Urate (umol/L)			0.915			0.879
Mean (SD)	317.60 (82.60)	317.93 (77.64)		312.34 (93.41)	312.13 (74.12)	
(Min, Max)	(167.00, 523.00)	(167.00, 541.00)		(172.00, 529.00)	(161.00, 500.00)	
Missing	1	4		4	24	
NfL (pg/mL)			0.107			0.537
Mean (SD)	11.84 (5.80)	13.17 (7.33)		12.84 (5.88)	14.60 (11.18)	
(Min, Max)	(2.76, 28.00)	(1.80, 76.60)		(3.67, 34.80)	(2.18, 131.00)	
Missing	10	18		8	40	

Cerebrospinal fluid biomarkers data of year 2–5 are not yet available. PD, Parkinson disease; ICBs, Impulse control behaviours; A-beta, A-beta 1–42; T-tau, total tau; P-tau181, tau phosphorylated at threonine 181; NfL, neurofilament light chain.

### 3.3. Cox regression analysis of individual risk factors of ICBs in PD subjects from baseline to year 5

**Figure 3:**
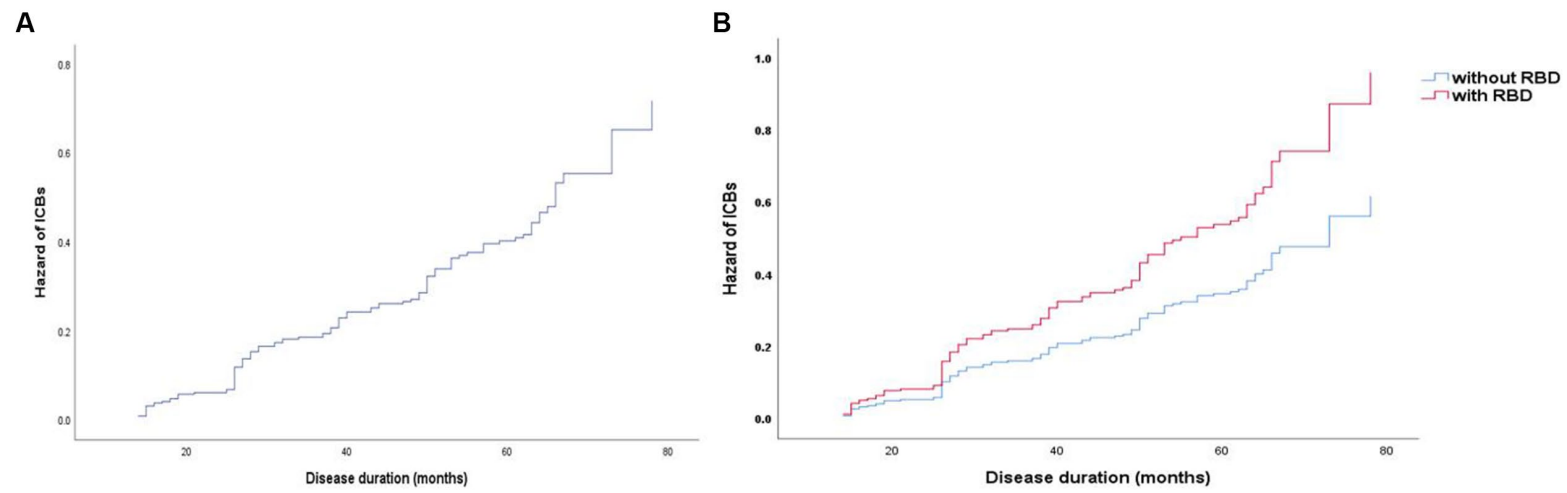
Survival curves of PD-ICBs. **(A)** Survival curves of PD-ICBs. **(B)** Survival curves of PD-ICBs with or without RBD. PD, Parkinson disease; ICBs, Impulse control behaviours; RBD, rapid eye movement sleep behaviour disorder.

**Figure 4:**
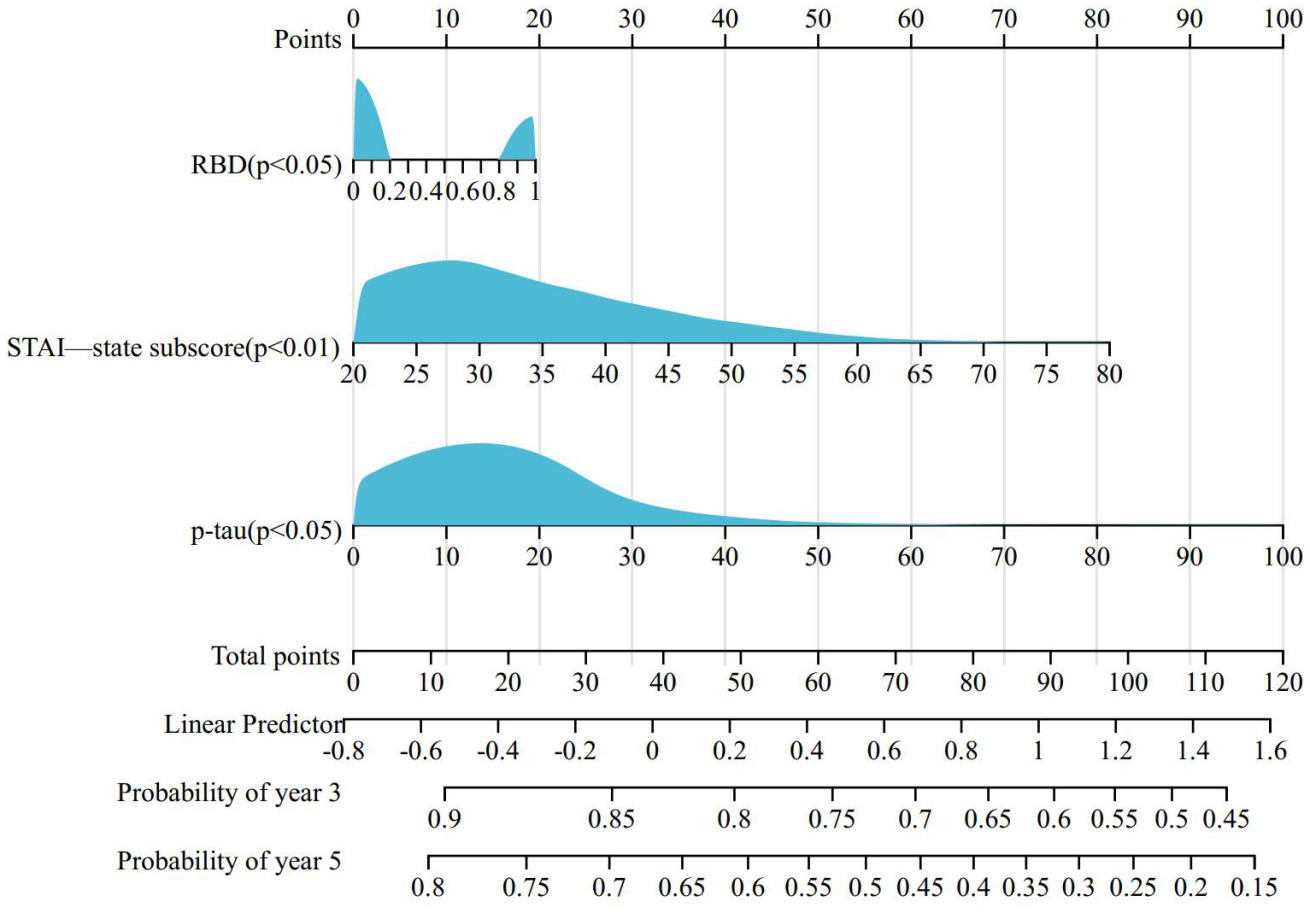
Nomogram for predicting prognosis of PD-ICBs. PD, Parkinson disease; ICBs, Impulse control behaviours; RBD, rapid eye movement sleep behaviour disorder.

To determine the baseline demographic, clinical, biological, imaging, and pharmacological variables that predict the incident development of ICBs over time, we evaluated the 318 participants with PD in this cohort who did not have ICBs at baseline. Among them, 116 participants (36.5%) subsequently developed ICBs. Results based on in-sample concordance and cross-validated prediction accuracy revealed that the best model included three variables: STAI-state subscore, RBD, and p-tau (Table 7; Supplementary Table S1). Additionally, estimated survival probabilities from the multivariate Cox regression models were obtained and are presented in Figures 3, 4.

**Table 7:** Cox regression analysis of individual risk factors of ICBs in PD subjects from baseline to year 5.

Variable	Univariate analysis		Multivariable analysis	
	HR (95% CI)	*p*-Value	HR(95% CI)	*p*-Value
Gender (male)	1.369 (0.914–2.048)	0.127	NS	NS
Age (years)	0.983 (0.965–1.001)	0.071	Not included	Not included
Age at PD onset (years)	0.983 (0.965–1.001)	0.067	NS	NS
Disease duration (month)	0.949 (0.915–0.984)	0.004	Not included	Not included
Education (years)	0.950 (0.892–1.011)	0.109	NS	NS
Family members with PD	1.306 (0.876–1.947)	0.190	NS	NS
MDS-UDPRS Part I	1.050 (1.006–1.095)	0.026	Not included	Not included
MDS-UDPRS Part II	1.015 (0.972–1.060)	0.506	–	–
MDS-UDPRS Part III	1.003 (0.983–1.023)	0.773	–	–
H&Y (stage > 1)	1.010 (0.702–1.455)	0.955	–	–
TD/non-TD classification	0.837 (0.565–1.241)	0.376	–	–
Tremor score	0.826 (0.460–1.486)	0.524	–	–
PIGD score	1.475 (0.648–3.359)	0.355	–	–
Side most affected (Left)	0.783 (0.537–1.140)	0.201	–	–
S&E	0.973 (0.944–1.002)	0.064	NS	NS
MoCA	1.005 (0.928–1.089)	0.903	–	–
GDS	1.064 (0.993–1.139)	0.077	NS	NS
STAI—state subscore	1.030 (1.013–1.047)	<0.001	1.027 (1.010–1.044)	0.002
STAI—trait subscore	1.030 (1.013–1.048)	0.001	NS	NS
RBD	1.641 (1.137–2.368)	0.008	1.555 (1.053–2.297)	0.027
ESS	1.045 (0.993–1.100)	0.089	NS	NS
SCOPA-AUT	1.018 (0.985–1.051)	0.290	–	–
Contralateral caudate	1.119 (0.784–1.598)	0.536	–	–
Ipsilateral caudate	1.185 (0.851–1.652)	0.315	–	–
Contralateral putamen	1.096 (0.522–2.303)	0.808	–	–
Ipsilateral putamen	1.298 (0.800–2.106)	0.291	–	–
A-beta (pg/mL)	0.999 (0.997–1.001)	0.262	–	–
T-tau (pg/mL)	1.000 (0.989–1.011)	0.959	–	–
p-tau (pg/mL)	1.016 (0.998–1.034)	0.076	1.021 (1.003–1.039)	0.021
Alpha-synuclein (pg/mL)	1.000 (1.000–1.000)	0.379	–	–
T-tau/A-beta	1.943 (0.093–40.751)	0.669	–	–
p-tau/A-beta	102.613 (2.548–4132.103)	0.014	Not included	Not included
p-tau/T-tau	1.690 (0.815–3.503)	0.158	Not included	Not included
Urate (umol/L)	1.001 (0.999–1.004)	0.301	–	–
NfL (pg/mL)	1.002 (0.976–1.028)	0.894	–	–
LEDD at DRT initiation (perΔ10 pts)	0.989 (0.974–1.005)	0.180	NS	NS
DRT delay from PD onset evaluations (month)	0.980 (0.965–0.996)	0.013	NS	NS

PD, Parkinson disease; ICBs, Impulse control behaviours; MDS-UPDRS, Movement Disorders Society-Unified Parkinson’s Disease Rating Scale; H&Y, Hoehn and Yahr; PIGD, Postural instability Gait Disorder; TD, tremor dominant; S&E, Modified Schwab and England Activities of Daily Living Scale; MoCA, Montreal Cognitive Assessment; GDS, Geriatric Depression Scale; STAI, State–Trait Anxiety Inventory; ESS, Epworth Sleepiness Scale; RBD, rapid eye movement sleep behaviour disorder; SCOPA-AUT, Scales for Outcomes in Parkinson’s Disease-Autonomic; QUIP, Questionnaire for Impulsive-Compulsive Disorders in Parkinson’s Disease; UPSIT, University of Pennsylvania Smell Identification Test; A-beta, A-beta 1–42; T-tau, total tau; P-tau181, tau phosphorylated at threonine 181; NfL, neurofilament light chain; LEDD, levodopa equivalent daily dose; DRT, dopamine replacement therapy; NS, not significant; ICBs, Impulse control behaviours; RBD, rapid eye movement sleep behaviour disorder. Age was not included in the multivariable model because Age at PD onset was already being considered. And p-tau/t-tau and p-tau/A-beta were not included in the multivariable model because p-tau was already being considered. Similarly, Total initial LEDD was not included in the multivariable model because Initial LEDD subtotal—non-dopamine agonists was already being considered.

## 4. Discussion

This international, multicenter study represents the largest reported longitudinal investigation of the incidence of ICBs and its associated clinical, imaging, and biological characteristics in patients with *de novo*, untreated PD at baseline. Furthermore, to our knowledge, this is the only study that has examined the association between biologics and ICBs in patients with PD. At baseline, there were no differences in the prevalence of ICBs between untreated patients with PD and HC. However, over time, the occurrence of ICBs increased among patients with PD while decreasing among HC. These findings are consistent with previous longitudinal studies that have examined ICBs in PD (11, 14, 21). In the PD cohort, there were notable differences in symptoms, particularly in non-motor symptoms, between patients with and without ICBs. This finding indicates that, in early untreated PD, the development of ICBs is influenced by factors beyond the presence of PD-related pathology alone. These factors may encompass various biological variables that affect neurochemical, neural network, and psychological systems as the disease advances, as well as clinical variables that impact disease progression, particularly the use of dopaminergic medications (2). In this study, we provide a thorough and systematic examination of the longitudinal changes in clinical and biological factors associated with PD-ICBs over a 5-year period. Additionally, we analyze the baseline clinical and biological predictors of ICB development in PD. The results obtained yield several intriguing insights into the pathophysiology of ICBs in PD.

Given that there are dynamic changes in ICBs in PD patients over the course of the observation, this is similar to other nonmotor symptoms of PD, which may disappear or recur. Supplementary Table S2 shows the data on the fluctuation of ICBs that we observed. We could find that the percentage of persistent ICBs+ ranged from 52.9% to 84.7% from the first to the fifth year, accounting for more than half of the cases. We recognize that therapeutic interventions and changes in a patient’s clinical course can affect their ICBs status over time.

In our cohort, demographic factors such as age, sex, education, and family history of PD, as well as disease characteristics including age of PD onset, disease duration, and severity of motor disability, were not found to be significant predictors of ICBs at baseline. This is in contrast to a previous cross-sectional study that identified male gender and younger age at onset of PD as risk factors for ICBs. The discrepancy in findings could be attributed to differences in study design, sample size, or other factors that may influence the development of ICBs in PD. Given this, we revisited the role of gender in our analysis after categorizing the dataset according to the classification of early-onset Parkinson’s disease (EOPD). That is, we performed a subgroup analysis of the dataset, taking into account the EOPD± classification. Interestingly, we observed no significant gender differences from baseline to year 5 in the EOPD+ subgroup, whether patients had ICBs or not. In contrast, within the EOPD− subgroup, it was only in the fifth year that the proportion of men in the ICBs+ group surpassed that in the ICBs− group (Supplementary Table S3). This suggests that the impact of gender on the occurrence of ICBs in non-EOPD patients may become more pronounced in the later stages of the disease, although further validation is required. Further research is needed to better understand the complex interplay of these factors in relation to ICBs in PD (15).

We speculate that the lack of significant association between ICBs and motor impairment measured in Part III of the MDS-UPDRS or other disease characteristics (such as H&Y stage, motor complications, clinical subtype, tremor score, PIGD score, and side most affected) may be due to the early stage of PD in our cohort and the possibility of interactions arising during disease progression or drug interventions. These factors were also not reported as significant predictors of ICBs in previous studies. However, we observed significant associations between ICBs and the neuro-psychiatric and non-motor symptoms assessed in Part I and the patient-reported experiences of daily living assessed in Part II of the MDS-UPDRS. This is not surprising, as previous studies have identified numerous non-motor symptoms as risk factors for ICBs in PD. These findings suggest that non-motor symptoms captured in Part I and Part II of the MDS-UPDRS may play a more prominent role in the development of ICBs in early-stage PD patients (3, 15, 16, 38). Considering that patients with EOPD may be more prone to the development of dyskinesia (39), we made the necessary distinction between two further distinct categories: EOPD+ (age at PD onset ≤50) and EOPD− (age at PD onset >50) (Supplementary Table S4). Our analysis revealed a higher percentage of EOPD+ individuals in the first year among patients with ICB+, whereas no significant difference was observed in the subsequent 4 years. This observation may also explain why the MDS-UPDRS Part IV scores were higher in the first year for patients with ICBs+ and then declined in the subsequent 4 years.

Our findings, in line with previous research, support the association between ICBs and anxiety in PD (38). This result is supported by a large number of neuroimaging studies with altered striatum and amygdala-orbital frontal cortex (OFC) circuits in patients with ICDs and anxiety (40–42). This association may suggest a biological basis, potentially involving noradrenergic and serotonergic structures that regulate mood. These neurochemical systems could play a role in the onset of ICBs. Additionally, it is possible that neuropsychological mechanisms are also involved in this association. Further investigation is needed to elucidate the specific pathways and mechanisms underlying the relationship between ICBs, anxiety, and the neurobiological and neuropsychological factors in PD (2). The diagnostic process of PD can indeed induce anxiety in patients, leading to possible excessive psychological distress. These psychological stresses may further increase patients’ susceptibility to developing ICBs. This association between anxiety and ICBs has been supported by previous research (38). However, in contrast to anxiety, our study did not find baseline depression levels to be a significant risk factor for the development of ICBs in early PD patients. It is important to note that the relationship between depression and ICBs may vary at different stages of the disease. While our study did not find a significant association between baseline depression and ICBs, there was a strong significant relationship between the two at the 5-year follow-up. This finding is inconsistent with a longitudinal study that reported an increased risk of ICBs later in the disease for individuals diagnosed with depression shortly after PD diagnosis (16). Indeed, the inconsistent result regarding the association between depression and the risk of developing ICBs in PD could be attributed to several factors. One possible explanation is the variation in the definition and assessment of depression used in different studies. Differences in the diagnostic criteria, assessment tools, and time points of assessment may contribute to the inconsistent findings. Additionally, the population characteristics and disease stage of the included participants could also play a role in the disparate results. In our study, we focused on early PD patients, whereas the longitudinal study that showed an association between depression and increased ICBs risk later in the disease may have included individuals with more advanced PD. It is possible that the impact of depression on ICBs risk varies throughout the course of the disease, with depression representing a greater risk factor in advanced stages of PD. Furthermore, it is noteworthy that neuroimaging studies have also provided evidence for a relationship. Namely, the dorsolateral prefrontal cortex (DLPFC) is altered in patients with ICDs, and this region is important as a key neural locus for depression (40, 41, 43). While these factors may partially explain the conflicting results, it is clear that further investigation is needed to fully understand the relationship between depression and ICBs in PD. Considering the potential significance of depression as a risk factor, it is important to incorporate the assessment of depression when screening for risk factors associated with the development of ICBs in patients with PD. By doing so, we can gain a more comprehensive understanding of the factors contributing to the occurrence of ICBs and potentially identify individuals at higher risk who may benefit from early interventions or tailored management strategies.

The relationship between EDS and ICBs in early PD is intriguing and has not been systematically explored. A study found associations between poor sleep efficiency, restless legs symptoms, and increased daytime sleepiness with impulsivity in PD (44). The study suggested that daytime sleepiness may disrupt the prefrontal cortex, which is responsible for inhibitory control of impulsive behavior, or that it may amplify the reactivity of brain reward networks (45). In our study, EDS was not found to be a significant risk factor for the development of ICBs. However, there was a significant difference between EDS and ICBs at the 5-year follow-up. Our results suggest that EDS may not facilitate the occurrence of ICBs but rather that ICBs lead to the emergence of EDS, which needs further validation in subsequent studies. Furthermore, PD patients with ICBs had higher rates of mood disturbance, which could potentially impact sleep quality directly (46). However, the result regarding RBD was different. In our study, RBD was not clearly associated with the development of ICBs in early PD, whereas multivariable analysis suggested RBD as a risk factor for ICBs development. RBD emerged as the strongest predictor of incident ICBs, with a hazard ratio (HR) of 1.555 (95% confidence interval: 1.053–2.297). Another study based on the PPMI found a significant association between RBD and ICBs in cross-sectional analyses, as well as an increased risk for ICBs symptoms in RBD patients in longitudinal univariate analysis. However, after adjustment for covariates, only a trend toward an increased risk was observed (47). Therefore, patients with PD who have RBD should be alerted to the potential development of ICBs.

The association between ICBs and autonomic dysfunction has not been systematically explored in early PD. Similar to EDS, our study did not identify autonomic dysfunction as an independent risk factor for the development of ICBs, although a significant difference was observed between autonomic dysfunction and ICBs at the 5-year follow-up. We speculate that autonomic damage may contribute further to the development of ICBs in patients. Interestingly, a study found that autonomic dysfunction is associated with reduced amygdala grey matter volume (48), which is considered part of the anatomical substrate of ICBs (49). This could suggest a common underlying mechanism between ICBs and autonomic dysfunction. However, this finding is inconsistent with the data from Ricciardi et al. (50) which indicated that autonomic dysfunction was a predictor of ICBs. The disparity in results may be attributed to differences in the study population and methodology. While there appears to be some association between autonomic dysfunction and ICBs, further investigations are necessary to confirm the intrinsic relationship between the two factors.

Many previous studies have indicated that drugs, particularly dopaminergic agonists, play a significant role in the development of ICBs in PD. However, limited research has focused on ICBs in early PD patients without medication use. A previous study proposed that PD itself may not increase the risk of developing ICBs, suggesting that the higher prevalence of ICBs in the PD population is driven by PD medications or other treatments such as deep brain stimulation. They suggested that certain clinical and demographic variables, such as younger age and family or personal history of similar behaviors, might simply moderate the risk of ICB development (51). However, we remain skeptical about this conclusion and question whether there are biological factors associated with PD itself that directly or indirectly influence the occurrence of ICBs. To address these hypotheses and explore potentially relevant factors, we conducted a systematic analysis of the demographic, clinical, dopamine transporter (DaT) scan imaging, and biological characteristics of the PD cohort. In the third and fourth years of follow-up, we observed a higher usage of dopamine agonists in patients with ICBs. Although the difference decreased in the fifth year compared to the previous year, this may be attributed to result bias caused by patient dropout during follow-up and delayed initiation of medication in early-stage patients. Therefore, the conclusion regarding the association between dopamine agonist use and ICBs does not contradict previous studies. As our baseline study population was not yet on medication and patients were not consistently taking medications, we included evaluations of LEDD at dopamine replacement therapy (DRT) initiation and the delay of DRT initiation from PD onset for correction. These factors did not contribute to the occurrence of ICBs in early PD patients in our study.

The novelty of our current study lies in the analysis of the association between biomarkers and ICBs in early PD patients. Unfortunately, we did not find a significant link between presynaptic dopaminergic dysfunction, as measured by DaTscan, and the occurrence of ICBs. However, a previous study based on pilot data from the PPMI suggested that the availability of dopamine receptors in the right striatum consistently decreased in PD patients with ICBs (52). The discrepancy in findings may be attributed to differences in study design, evaluation criteria, and the selection of laterality. The precise biological relationship between presynaptic dopaminergic dysfunction and ICBs remains unclear.

Impulsivity and reward-based decision-making are mediated by a complex neural network involving interconnected mesocortical and mesolimbic circuits. One hypothesis is that a combination of pre-existing biological and genetic risk factors contributes to the relative preservation of dopamine receptor functions. An alternative hypothesis is that the relative preservation of dopamine receptors may lead to impaired inhibition of impulsivity, potentially contributing to the development of ICBs in PD patients. However, in our study conducted in early PD patients, we did not find evidence supporting these hypotheses. Further exploration of these relationships in advanced-stage PD patients is warranted.

Although no significant association was found between ICBs and CSF biomarkers, such as NfL or urate, at baseline and year 1, our multifactorial analysis suggested that levels of p-tau in CSF may serve as an independent risk factor for the development of ICBs. This finding is novel and has not been proposed before. However, it’s important to note that the CSF data in our study were limited to the first year, and further confirmation of this association in patients with longer disease duration is necessary. Given the role of p-tau as a biomarker of neurodegeneration, it will be of great interest to investigate in future analyses whether CSF p-tau values increase at higher rates in individuals with ICBs. This could provide insights into the potential relationship between ICBs and the progression of neurodegenerative processes. Further studies with longer-term follow-up and more comprehensive CSF biomarker assessments are warranted to explore this potential link.

## 5. Limitations

Despite the comprehensive exploration of factors associated with ICBs in early PD, there are several limitations to acknowledge in our study. Firstly, the assessment of ICBs relied on a short version of the QUIP, which primarily screened for the presence of ICBs but did not provide a detailed evaluation of the severity of symptoms. This limited our ability to capture the full spectrum of ICB-related symptoms and their impact on patients. Additionally, we acknowledge that there are pending longitudinal data analyses for CSF and imaging data beyond the initial years of follow-up. The analysis of CSF data from years 2 to 5 and imaging data from years 3 and 5 on this cohort is still underway. These data could provide valuable insights into the long-term associations between these factors and the development of ICBs in early PD. These limitations highlight the need for further studies that employ more comprehensive and detailed assessment methods for ICBs and include longer-term follow-up to better understand the complexity of ICBs in PD.

## 6. Conclusion

In conclusion, our study, which included the largest longitudinal case–control cohort of *de novo* unmedicated PD patients, provides valuable insights into the clinical and biological factors associated with ICBs in PD. We observed that the prevalence of ICBs increases over time in PD patients, while it decreases over time in the healthy control group. Our findings support previous research indicating that ICBs are associated with comorbid conditions such as depression and anxiety, as well as autonomic dysfunction, EDS, and the use of dopaminergic medications, particularly dopamine agonists. Moreover, our study identified several predictors of the incident development of ICBs in early PD. These predictors include anxiety, RBD, and elevated levels of p-tau in CSF. These findings suggest that these factors may play a role in the pathogenesis of ICBs in PD and could potentially be used as indicators for the development of preventive strategies or targeted interventions. Overall, our study contributes to the understanding of ICBs in PD and highlights the importance of considering both clinical and biological factors in assessing the risk and progression of ICBs in early PD patients. Further research is needed to validate these findings and to explore potential mechanisms underlying the observed associations.

## Data availability statement

The datasets presented in this study can be found in online repositories. The names of the repository/repositories and accession number(s) can be found in the article/Supplementary material.

## Ethics statement

The studies involving humans were approved by Ethics Committee of Xinhua Hospital Affiliated to Shanghai Jiao Tong University School of Medicine. The studies were conducted in accordance with the local legislation and institutional requirements. The participants provided their written informed consent to participate in this study.

## Author contributions

XZ: Conceptualization, Data curation, Formal analysis, Visualization, Writing – original draft. JG: Supervision, Validation, Writing – review & editing. NW: Supervision, Validation, Resources, Writing – review & editing. YW: Supervision, Validation, Resources, Writing – review & editing. LS: Supervision, Validation, Resources, Writing – review & editing. ZL: Conceptualization, Funding acquisition, Resources, Supervision, Writing – review & editing. YZ: Conceptualization, Funding acquisition, Methodology, Resources, Visualization, Writing – review & editing.

## Footnotes

^1^http://www.ppmi-info.org
