# Supplementary material for: Assessing impulse control behaviors in early Parkinson’s disease: a longitudinal study
Source: Front Neurol. 2023 Oct 25;14:1275170. doi: 10.3389/fneur.2023.1275170 (PMC10634396; doi:10.3389/fneur.2023.1275170)
Supplement: Supplementary file 1 [file Data_Sheet_1.doc]

| **Supplementary material table 1 Cox regression analysis of individual risk factors of ICBs in PD subjects from baseline to year 5** | | | | | | | | | | | | | | |
| --- | --- | --- | --- | --- | --- | --- | --- | --- | --- | --- | --- | --- | --- | --- |
| **Omnibus Tests of Model Coefficients^d^** | | | | | | | | | | | | | | |
| Step | -2 Log Likelihood | | Overall (score) | | | Change From Previous Step | | | Change From Previous Block | | | | | |
|  |  |  | Chi-square | df | Sig. | Chi-square | df | Sig. | Chi-square | | df | | | Sig. |
| 1^a^ | 1141.148 | | 11.248 | 1 | 0.001 | 10.093 | 1 | 0.001 | 10.093 | | 1 | | | 0.001 |
| 2^b^ | 1137.696 | | 15.202 | 2 | 0.000 | 3.452 | 1 | 0.063 | 13.545 | | 2 | | | 0.001 |
| 3^c^ | 1132.913 | | 19.945 | 3 | 0.000 | 4.783 | 1 | 0.029 | 18.328 | | 3 | | | 0.000 |
| a. Variable(s) Entered at Step Number 1: STAI—state subscore | | | | | | | | | | | | | | |
| b. Variable(s) Entered at Step Number 2: p-Tau181P | | | | | | | | | | | | | | |
| c. Variable(s) Entered at Step Number 3: RBD | | | | | | | | | | | | | | |
| d. Beginning Block Number 1. Method = Forward Stepwise (Likelihood Ratio) | | | | | | | | | | | | | | |
| **Variables in the Equation** | | | | | | | | | | | | | | |
|  | | | | B | SE | Wald | df | Sig. | Exp(B) | 95.0% CI for Exp(B) | | | | |
|  |  |  |  |  |  |  |  |  |  | Lower | | Upper | | |
| Step 1 | | STAI—state subscore | | 0.028 | 0.008 | 11.126 | 1 | 0.001 | 1.029 | 1.012 | | | 1.046 | |
| Step 2 | | STAI—state subscore | | 0.029 | 0.008 | 11.579 | 1 | 0.001 | 1.029 | 1.012 | | | 1.046 | |
|  |  | p-Tau181P | | 0.018 | 0.009 | 4.032 | 1 | 0.045 | 1.018 | 1.000 | | | 1.036 | |
| Step 3 | | STAI—state subscore | | 0.027 | 0.009 | 9.716 | 1 | 0.002 | 1.027 | 1.010 | | | 1.044 | |
|  |  | RBD | | 0.442 | 0.199 | 4.922 | 1 | 0.027 | 1.555 | 1.053 | | | 2.297 | |
|  |  | p-Tau181P | | 0.021 | 0.009 | 5.321 | 1 | 0.021 | 1.021 | 1.003 | | | 1.039 | |

| **Supplementary material table 2 Fluctuations in the occurrence of ICBs in PD patients** | | | | | | |
| --- | --- | --- | --- | --- | --- | --- |
| **Time** | **Baseline** | **Year 1** | **Year 2** | **Year 3** | **Year 4** | **Year 5** |
| Total | 401 | 362 | 362 | 360 | 340 | 311 |
| Total of ICBs+ | 83(20.7%) | 51(14.1%) | 76(21.0%) | 86(23.9%) | 92(27.1%) | 85(27.3%) |
| newly occurring ICBs+ | 83(20.7%) | 24(6.6%) | 33(9.1%) | 24(6.7%) | 23(6.8%) | 13(4.2%) |
| Previously ICBs+ | / | 27(7.5%) | 43(11.9%) | 62(17.2%) | 69(20.3%) | 72(23.1%) |
| Percentage of persistent ICBs+ | / | 27/51(52.9%) | 43/76(56.6%) | 62/86(72.1%) | 69/92(75.0%) | 72/85(84.7%) |

| **Supplementary material table 3 Gender of EOPD patients or non EOPD patients with and without ICBs** | | | | | | | | | | | | | | | | | | |
| --- | --- | --- | --- | --- | --- | --- | --- | --- | --- | --- | --- | --- | --- | --- | --- | --- | --- | --- |
| **Gender of EOPD patients with and without ICBs** | | | | | | | | | | | | | | | | | | |
| **Variable** | **Baseline** | | | **Year 1** | | | **Year 2** | | | **Year 3** | | | **Year 4** | | | **Year 5** | | |
|  | **PD ICBs+**  **n = 20** | **PD ICBs-**  **n = 58** | **p** | **PD ICBs+**  **n = 15** | **PD ICBs-**  **n = 56** | **p** | **PD ICBs+**  **n =17** | **PD ICBs-**  **n = 55** | **p** | **PD ICBs+**  **n = 22** | **PD ICBs-**  **n = 49** | **p** | **PD ICBs+**  **n = 22** | **PD ICBs-**  **n = 48** | **p** | **PD ICBs+**  **n = 20** | **PD ICBs-**  **n = 47** | **p** |
| **Gender** |  |  | 0.978 |  |  | 0.940 |  |  | 0.830 |  |  | 0.596 |  |  | 0.248 |  |  | 0.609 |
| **Male** | 12  (60.00%) | 35  (60.34%) |  | 9  (60.00%) | 33  (58.93%) |  | 11  (64.71%) | 34  (61.81%) |  | 12  (54.55%) | 30  (61.22%) |  | 11  (50.00%) | 31  (64.58%) |  | 11  (55.00%) | 29  (61.70%) |  |
| **Female** | 8  (40.00%) | 23  (39.66%) |  | 6  (40.00%) | 23  (41.07%) |  | 6  (35.29%) | 21  (38.19%) |  | 10  (45.45%) | 19  (38.78%) |  | 11  (50.00%) | 17  (35.42%) |  | 9  (45.00%) | 18  (38.30%) |  |
| **Missing** | 0 | 0 |  | 0 | 0 |  | 0 | 0 |  | 0 | 0 |  | 0 | 0 |  | 0 | 0 |  |
| **Gender of non-EOPD patients with and without ICBs** | | | | | | | | | | | | | | | | | | |
| **Variable** | **Baseline** | | | **Year 1** | | | **Year 2** | | | **Year 3** | | | **Year 4** | | | **Year 5** | | |
|  | **PD ICBs+**  **n = 63** | **PD ICBs-**  **n = 260** | **p** | **PD ICBs+**  **n = 36** | **PD ICBs-**  **n = 255** | **p** | **PD ICBs+**  **n =59** | **PD ICBs-**  **n = 231** | **p** | **PD ICBs+**  **n = 64** | **PD ICBs-**  **n = 225** | **p** | **PD ICBs+**  **n = 70** | **PD ICBs-**  **n = 200** | **p** | **PD ICBs+**  **n =65** | **PD ICBs-**  **n = 179** | **p** |
| **Gender** |  |  | 0.736 |  |  | 0.506 |  |  | 0.433 |  |  | 0.200 |  |  | 0.200 |  |  | **0.004** |
| **Male** | 41  (65.07%) | 175  (67.31%) |  | 26  (72.22%) | 170  (66.67%) |  | 42  (71.19%) | 152  (65.80%) |  | 47  (73.44%) | 146  (64.89%) |  | 52  (74.29%) | 132  (66.00%) |  | 54  (83.08%) | 114  (63.69%) |  |
| **Female** | 22  (34.93%) | 85  (32.69%) |  | 10  (27.78%) | 85  (33.33%) |  | 17  (28.81%) | 79  (34.20%) |  | 17  (26.56%) | 79  (35.11%) |  | 18  (25.71%) | 68  (34.00%) |  | 11  (16.92%) | 65  (36.31%) |  |
| **Missing** | 0 | 0 |  | 0 | 0 |  | 0 | 0 |  | 0 | 0 |  | 0 | 0 |  | 0 | 0 |  |

| **Supplementary material table4 MDS-UDPRS part IV and Percentage of EOPD of PD patients with and without ICBs** | | | | | | | | | | | | | | | | | | |
| --- | --- | --- | --- | --- | --- | --- | --- | --- | --- | --- | --- | --- | --- | --- | --- | --- | --- | --- |
| **Variable** | **Baseline** | | | **Year 1** | | | **Year 2** | | | **Year 3** | | | **Year 4** | | | **Year 5** | | |
|  | **PD ICBs+**  **n = 83** | **PD ICBs-**  **n = 318** | **p** | **PD ICBs+**  **n = 51** | **PD ICBs-**  **n = 311** | **p** | **PD ICBs+**  **n =76** | **PD ICBs-**  **n = 286** | **p** | **PD ICBs+**  **n = 86** | **PD ICBs-**  **n = 274** | **p** | **PD ICBs+**  **n = 92** | **PD ICBs-**  **n = 248** | **p** | **PD ICBs+**  **n = 85** | **PD ICBs-**  **n = 226** | **p** |
| **MDS-UDPRS part IV** |  |  | NA |  |  | 0.029 |  |  | 0.423 |  |  | 0.516 |  |  | 0.577 |  |  | 0.282 |
| **Mean (SD)** | NA | NA |  | 0.94  (1.69) | 0.25  (0.97) |  | 0.52  (1.34) | 0.72  (1.85) |  | 0.87  (1.77) | 1.04  (2.00) |  | 1.42  (2.54) | 1.61  (2.66) |  | 1.83  (3.02) | 2.24  (2.93) |  |
| **(Min, Max)** |  |  |  | (0.00,  5.00) | (0.00,  7.00) |  | (0.00,  6.00) | (0.00,  11.00) |  | (0.00,  6.00) | (0.00,  11.00) |  | (0.00,  13.00) | (0.00,  12.00) |  | (0.00,  16.00) | (0.00,  17.00) |  |
| **Missing** |  |  |  | 18 | 127 |  | 14 | 42 |  | 9 | 19 |  | 7 | 10 |  | 2 | 12 |  |
| **EOPD** |  |  | NA |  |  | 0.021 |  |  | 0.744 |  |  | 0.895 |  |  | 0.410 |  |  | 0.257 |
| **Positive (age at PD onset≤50)** | NA | NA |  | 11  (21.57％) | 32  (10.30％) |  | 7  (9.21％） | 30  (10.49％） |  | 6  (6.98％) | 18  (6.57％) |  | 7  (7.61％) | 13  (5.24％) |  | 7  (8.24％) | 11  (4.87％) |  |
| **Negative(age at PD onse>50)** | NA | NA |  | 40  (78.43％) | 279  (89.70％) |  | 69  (90.79％） | 256  (89.51％） |  | 80  (93.02％) | 256  (93.43％) |  | 85  (92.39％) | 235  (94.76％) |  | 78  (91.76％) | 215  (95.13％) |  |
| **Missing** |  |  |  | 0 | 0 |  | 0 | 0 |  | 0 | 0 |  | 0 | 0 |  | 0 | 0 |  |

| **Supplementary material table 5 Cognitive Function in PD patients with and without ICBs** | | | | | | | | | | | | | | | | | | |
| --- | --- | --- | --- | --- | --- | --- | --- | --- | --- | --- | --- | --- | --- | --- | --- | --- | --- | --- |
| **Variable** | **Baseline** | | | **Year 1** | | | **Year 2** | | | **Year 3** | | | **Year 4** | | | **Year 5** | | |
|  | **PD ICBs+**  **n = 83** | **PD ICBs-**  **n = 318** | **p Value** | **PD ICBs+**  **n = 51** | **PD ICBs-**  **n = 311** | **p Value** | **PD ICBs+**  **n =76** | **PD ICBs-**  **n = 286** | **p Value** | **PD ICBs+**  **n = 86** | **PD ICBs-**  **n = 274** | **p Value** | **PD ICBs+**  **n = 92** | **PD ICBs-**  **n = 248** | **p Value** | **PD ICBs+**  **n = 85** | **PD ICBs-**  **n = 226** | **p Value** |
| **Cognitive Function** |  |  | 0.458 |  |  | 0.925 |  |  | 0.651 |  |  | 0.662 |  |  | 0.896 |  |  | 0.503 |
| **MOCA(＞26)** | 52  (62.7%) | 213  (67.0%) |  | 28  (54.9%) | 168  (54.2%) |  | 39  (51.3%) | 154  (54.2%) |  | 49  (57.6%) | 150  (54.9%) |  | 55  (60.4%) | 150  (61.2%) |  | 55  (65.5%) | 138  (61.3%) |  |
| **MOCA(**≤**26)** | 31  (37.3%) | 105  (33.0%) |  | 23  (45.1%) | 142  (45.8%) |  | 37  (48.7%) | 130  (45.8%) |  | 36  (42.4%) | 123  (45.2%) |  | 36  (39.6%) | 95  (388.8%) |  | 29  (34.5%) | 87  (38.7%) |  |
| **Missing** | 0 | 0 |  | 0 | 1 |  | 0 | 2 |  | 1 | 1 |  | 1 | 3 |  | 1 | 1 |  |
